# Patients with sepsis exhibit increased mitochondrial respiratory capacity in peripheral blood immune cells

**DOI:** 10.1186/cc12831

**Published:** 2013-07-24

**Authors:** Fredrik Sjövall, Saori Morota, Johan Persson, Magnus J Hansson, Eskil Elmér

**Affiliations:** 1 Mitochondrial Pathophysiology Unit, Department of Clinical Sciences, Lund University, Lund, Sweden; 2 Intensive Care Unit 4131, Copenhagen University Hospital, Rigshospitalet, Copenhagen, Denmark; 3 Departments of Anesthesiology and Intensive Care, Skåne University Hospital, Lund, Sweden; 4 Clinical Physiology, Skåne University Hospital, Lund, Sweden; 5 Clinical Neurophysiology, Skåne University Hospital, Lund, Sweden

## Abstract

**Introduction:**

In sepsis, mitochondria have been associated with both initial dysfunction and subsequent upregulation (biogenesis). However, the evolvement of mitochondrial function in sepsis over time is largely unknown, and we therefore investigated mitochondrial respiration in peripheral blood immune cells (PBICs) in sepsis patients during the first week after admission to the intensive care unit (ICU).

**Methods:**

PBICs from 20 patients with severe sepsis or septic shock were analyzed with high-resolution respirometry 3 times after admission to the ICU (within 48 hours, days 3 to 4 and days 6 to 7). Mitochondrial DNA (mtDNA), cytochrome *c* (Cyt *c*), and citrate synthase (CS) were measured as indicators of cellular mitochondrial content.

**Results:**

In intact PBICs with endogenous substrates, a gradual increase in cellular respiration reached 173% of controls after 1 week (*P* = 0.001). In permeabilized cells, respiration using substrates of complex I, II, and IV were significantly increased days 1 to 2, reaching 137%, 130%, and 173% of controls, respectively. In parallel, higher levels of CS activity, mtDNA, and Cyt *c* content in PBICs (211%, 243%, and 331% of controls for the respective indicators were found at days 6 to 7; *P* < 0.0001). No differences in respiratory capacities were noted between survivors and nonsurvivors at any of the time points measured.

**Conclusions:**

PBICs from patients with sepsis displayed higher mitochondrial respiratory capacities compared with controls, due to an increased mitochondrial content, as indicated by increased mitochondrial DNA, protein content, and enzyme activity. The results argue against mitochondrial respiratory dysfunction in this type of cells in sepsis.

## Introduction

Sepsis is one of the leading causes of admission to the intensive care unit (ICU). No definitive treatment exists, and despite advancement in supportive therapies, mortality is still high. Today, a minority of patients succumb in the initial phase of acute shock, and rather enters the ensuing more prolonged phase of intensive care, characterized by multiple organ failure (MOF) and the need for organ-supportive therapies. Today, the primary cause of death from sepsis is due to unresolving MOF with withdrawal of supportive therapies [1,2]. The initial phase of sepsis is dominated by the systemic inflammatory response syndrome (SIRS) [3]. This proinflammatory response will gradually convert to an antiinflammatory response, denoted the compensatory antiinflammatory response syndrome (CARS) [4]. In this latter stage, immune cell function of all major cell lines, such as dendritic cells, lymphocytes, and neutrophils, has been suggested to be downregulated, leading to an immunoparalysis, or anergy, which could leave the patient more vulnerable to deleterious secondary infections [5-8].

Mitochondrial dysfunction has been implicated as a causative mechanism for reduced activity of immune cells in sepsis. Several investigations have demonstrated reduced function of different aspects of mitochondrial respiratory activity of peripheral blood immune cells (PBICs) in the early disease stages of sepsis patients admitted to the ICU [9-11]. The results are, however, somewhat divergent, probably reflecting differences in study population, experimental setup, and what mitochondria-specific markers have been chosen for normalization of respiration to cellular content of mitochondria. The evolution of mitochondrial respiratory function in PBICs in the later stages of sepsis is still largely unknown. Also, it is clear from several studies that sepsis induces a biogenesis response in which mitochondrial mass, number, and/or function increases after the initial phase of the septic event [12-14].

As PBICs play a central role in the septic syndrome and with the obvious dynamic changes occurring in the course of sepsis, we were interested in exploring the evolution of mitochondrial respiratory function in human PBICs and its relation to outcome.

The specific aims of the present study were to investigate PBIC mitochondrial respiration, by using high-resolution respirometry, during the first week of sepsis and to evaluate the response in relation to three different markers of mitochondrial content. Also, we evaluated whether mitochondrial respiration in PBICs differed between survivors and nonsurvivors.

## Materials and methods

### Patients

The study was approved by the scientific ethical committee of Copenhagen County, Denmark (H-C-2008-023), and the regional ethical review board of Lund, Sweden (113/2008, 79/2011, 89/2011). Patients were recruited from the intensive care units (ICUs) of Lund University Hospital and Copenhagen University Hospital, Rigshospitalet. Written, informed consent was obtained from the patient or next of kin. In Denmark, consent from the patient’s primary health care physician was also required if the patient was not able to consent. The diagnosis of sepsis was established by definitions previously described [3], and severe sepsis was defined as sepsis complicated with at least one organ failure, defined as sequential organ failure score (SOFA) ≥2. Septic shock was defined as circulatory failure requiring inotropic support to maintain a systolic blood pressure >90 mm Hg or mean arterial pressure >65 mm Hg, after adequate fluid resuscitation. Patients were included within 48 hours after their admission to the ICU. For patients transferred from other hospitals, the diagnosis of sepsis should not have been made more than 24 hours before ICU admission. Patients with known mitochondrial disease, hematologic malignancy, or who were pregnant were excluded. Blood samples were taken at three different time points during the first week after admission to the ICU: within the first 48 hours (days 1 to 2), at days 3 to 4, and at days 6 to 7. Thirty-one healthy individuals served as controls, from whom blood samples were taken after written and informed consent was acquired.

### Sample preparation

In patients, a maximal volume of 40 ml of blood was drawn from an existing arterial line in K_2_EDTA tubes (Vacuette®, Greiner Bio-One GmbH, Kremmünster, Austria). In controls, blood samples were taken via venous puncture in K_2_EDTA tubes. PBICs were isolated from whole blood by Ficoll gradient centrifugation [15]. After washing in normal saline, cells were resuspended in 200 to 400 μl of saline, depending on yield, together with 50 to 100 μl of the subject’s own plasma. Final concentration was determined in a Swelab Alfa hematology analyzer (Boule Medical AB, Stockholm, Sweden). Median cell count after preparation was 100 × 10^6^ cells/ml (range, 15 to 280). Respirometric measurements were performed within 5 hours of sampling. The analyzed contents from the respirometry chamber were stored frozen until further use.

### High-resolution respirometry

PBICs were placed in the 2-ml oxygraph chamber at a final concentration of 2.5 to 5 × 10^6^ cells/ml. In intact cells, respiration media consisted of the subject’s own plasma, and for permeabilized cells, a respiration media containing sucrose, 110 m*M*; HEPES, 20 m*M*; taurine, 20 m*M*; K-lactobionate, 60 m*M*; MgCl_2,_ 3 m*M*; KH_2_PO_4_, 10 m*M*; EGTA, 0.5 m*M*; BSA, 1 g/L; pH 7.1 (MiR05) [16]. Measurements were performed at a constant temperature of 37°C in a high-resolution oxygraph (Oxygraph-2k; Oroboros Instruments, Innsbruck, Austria). Oxygen concentration (micromolar) and oxygen flux (negative time derivative of oxygen concentration; pmol O_2_ × s^-1^ × 10^-6^ cells) was recorded with DatLab software 4.3 (Oroboros Instruments, Innsbruck, Austria). All experiments were performed at an oxygen concentration >50 μ*M* O_2._ Calibration at air saturation was performed each day. Instrumental background was measured in a separate set of experiments and automatically corrected for in the ensuing experiments, according to the manufacturer’s instructions.

Oxygen concentration was automatically calculated from barometric pressure and solubility factors that were set to 1.0 for water, 0.92 for MiR05, and 0.89 for plasma [17].

Two different protocols were used, one in intact cells and one in permeabilized cells [13,18]. In intact cells, respiration is maintained by endogenous plasma-derived substrates only. Intact cells were suspended in the subject’s own plasma and were allowed to stabilize at so-called routine respiration. Oligomycin (1 μg/ml) was then added to induce a state 4-like respiration (LEAK or State 4o) in which respiration is primarily related to leakage of protons over the inner mitochondrial membrane. Maximal oxygen flux (as supported by plasma-derived substrates) was subsequently obtained by stepwise (20 to 40 μ*M*) titration of the uncoupler FCCP. Finally, complex I and complex III were inhibited by adding rotenone (2 μ*M*) and subsequently antimycin-A (1 μg/ml). The residual oxygen flux after antimycin addition was subtracted from the steady-state respiration values.

The second protocol was performed in permeabilized cells. After cells had stabilized at routine respiration, the plasma cell membrane was permeabilized by adding digitonin (6 μg/1 × 10^6^ cells; the optimal concentration of digitonin was evaluated in a different set of experiments; data not shown). Simultaneously, the complex I substrates malate and pyruvate (5 m*M*, respectively) were added.

The ensuing addition of excess ADP (1 m*M*) stimulated respiration and represents maximal oxidative phosphorylation capacity (OXPHOS or State 3) for that specific, NADH-linked, substrate combination. With the subsequent addition of glutamate (5 m*M*), pyruvate dehydrogenase is bypassed, and maximal NADH-linked complex I respiration, OXPHOS_CI_, was obtained. The subsequent addition of succinate (10 m*M*) stimulated respiration further because of convergent input of electrons through both complex I and complex II via the Q-junction, OXPHOS_CI+II_.

After maximal OXPHOS_CI+II_ capacity, ATP-synthase was inhibited by oligomycin (1 μg/ml) to evaluate LEAK_CI+II_ (State 4o). Maximal electron transportation through respiration without oxidative phosphorylation, ETS_CI+II_, was evaluated by stepwise (2 to 4 μ*M*) titration of FCCP. Uncoupled complex II-linked respiration, ETS_CII,_ was evaluated by adding rotenone, and subsequently, the ETS was inhibited by antimycin-A. At this point, reoxygenation of the oxygraph chamber was performed to a level of 160 to 180 μ*M* O_2._ The activity of complex IV was evaluated by adding N,N,N’,N’-*tert-*methyl-*p*-phenyldiamine (TMPD 0.5 m*M*), an electron donor to complex IV. Because of the high level of autoxidation of TMPD, sodium azide (10 m*M*), an inhibitor of complex IV, was added and the difference between the two levels obtained was calculated as the specific complex IV activity. Control ratios were derived from maximal FCCP-stimulated respiration or maximal oxidative respiration divided by LEAK respiration.

### Analysis of mitochondrial DNA

Mitochondrial DNA (mtDNA) was measured, as previously described [19], with certain modifications. Frozen samples (oxygraph chamber content after respiration measurements in MiR05) were thawed, sonicated, and subsequently diluted 500 times in a buffer containing 10 m*M* Tris-HCl, 1 m*M* EDTA, salmon sperm DNA, 1 ng/μl, pH 8.0. Ten microliters of this dilution were amplified in a 25-μl PCR reaction containing 1 × Power SYBR® Green PCR Master Mix by using a StepOnePlus™ Real-Time PCR System (Applied Biosystems Inc., Foster City, CA, USA) and 100 n*M* of each primer (Eurofins MWG Operon, GmbH, Ebersberg, Germany). The primers targeted the human mitochondrial *COX-1* gene (forward: CCC CTG CCA TAA CCC AAT ACC A, reverse: CCA GCA GCT AGG ACT GGG AGA GA). The threshold cycle (Ct) values were related to a standard curve by using cloned PCR products (kindly provided by P. Schjerling, University of Copenhagen, Denmark). Samples were analyzed in duplicate.

### Cytochrome *c* determination

Human cytochrome *c* (Cyt *c*) content was quantified by using an immunoassay kit (DCTC0, Quantikine®, R&D Systems, Abingdon, UK). Frozen samples (same as described earlier) were thawed and sonicated and subsequently processed according to the manufacturer’s instructions.

### Citrate synthase determination

A commercially available kit (Citrate Synthase Assay Kit, CS 0720; Sigma), was used according to manufacturer’s instructions, to determine citrate synthase (CS) activity in the frozen samples. Data are expressed as arbitrary units (a.u.).

### Cytokine measurement

The levels of the proinflammatory cytokines tumor necrosis factor (TNF)-α, IL-1β, and IL-6 were analyzed with a multiplex sandwich immunoassay according to the manufacturer’s instructions (MSD® 96-well Multi-Spot®; Meso Scale Discovery, Gaithersburg, MD, USA). In brief, precoated 96-well plates were incubated with plasma samples for 2 hours. Subsequently, detection antibodies were added, and the plate incubated for another 2 hours. After washing, the plate was read with MSD Sector Imager®. Because the variation of the cytokine levels in the healthy controls was low, the number was restricted to 12 randomly selected subjects.

### Data analysis

Statistics were calculated by using Graph Pad Prism v. 5 (GraphPad Software Inc., La Jolla, CA, USA). Data were tested for normal distribution with the D’Agostino and Pearson omnibus normality test. Data are presented as mean ± SD if not indicated otherwise. Analysis between groups was performed by using the unpaired Student's *t* test. Differences between days were analyzed with repeated measures ANOVA and, when significant, paired Student's *t* tests between days were performed. The results were adjusted with Bonferroni correction for multiple tests. Correlations were performed by using linear regression. A *P* value of <0.05 was considered statistically significant.

## Results

We recruited 20 patients with severe sepsis or septic shock and 31 controls, whose characteristics are presented in Table 1. The patients included were predominantly female (13 of 20) with a median age of 70 years (IQR, 62 to 74.5 years). The primary sites of infection were soft tissue (nine of 20), abdominal (six of 20), chest (four of 20), and urinary tract (one of 20). Table 2 shows clinical characteristics of the sepsis patients during the first week in the ICU. Five patients (25%) died, and another five patients were discharged or transferred to other facilities within the first week leaving the patient cohort at days 3 to 4 with *n* = 15 and *n* = 10 at days 6 to 7. Noradrenaline was the preferred inotropic drug, with dobutamine added in some cases.

**Table 1:** Characteristics of patients and controls

	**Patients**	**Controls**
Age, years	70 (62–75)	31 (23–59)
Sex, female/male	13/7	15/16
SAPS II	50 (38–61)	
APACHE II	23 (18–30)	
Source of sepsis		
Soft tissue	9	
Abdomen	6	
Chest	4	
Urinary tract	1	
Severe sepsis/septic shock	1/19	
Outcome		
28-day mortality (%)	6 (30)	
90-day mortality (%)	7 (35)	

Data presented as median (IQR) or absolute values. SAPS, Simplified Acute Physiologic Score; APACHE, Acute Physiology and Chronic Health Evaluation score.

**Table 2:** Clinical characteristics of patients and controls at time of blood sampling

	**Day of sample 1**	**Day of sample 2**	**Day of sample 3**	**Controls**
SOFA	8.0 (6.5-11)	7.0 (3–10)	6.5 (4.8-9.8)	-
Noradrenaline	0.13 (0.045-0.52)	0.0 (0.0-0.08)	0.0 (0.0-0.09)	-
Dobutamine	4.7 (2.9-9.1)	2.0 (1.0-10)	-	-
IL-6	259 (112–1433)^a^	51 (30–80)^a^	26 (18–65)^a^	1.2 (1.0–2.3)
TNF-α	26 (10–44)^a^	11 (8.9-20)^a^	6.7 (5.2-8.7)^b^	3.7 (2.7-4.2)
IL-1β	2.1 (1.2-3.5)^a^	0.9 (0.6-1.3)	0.9 (0.7-1.2)	0.5 (0.2-0.6)

Data presented as median (IQR). SOFA*,* Sequential Organ Failure Assessment. Inotropic drugs are expressed as μg/kg/min, and cytokine levels as pg/ml. Patients receiving noradrenaline: sample 1, *n* = 19; sample 2, *n* = 6; sample 3, *n* = 3. Dobutamine: sample 1, *n* = 4; sample 2, *n* = 3; sample 3, *n* = 0. ^a^*P* < 0.001; ^b^*P* < 0.05, compared with controls.

### Cellular respiration in septic patients

**Figure 1:**
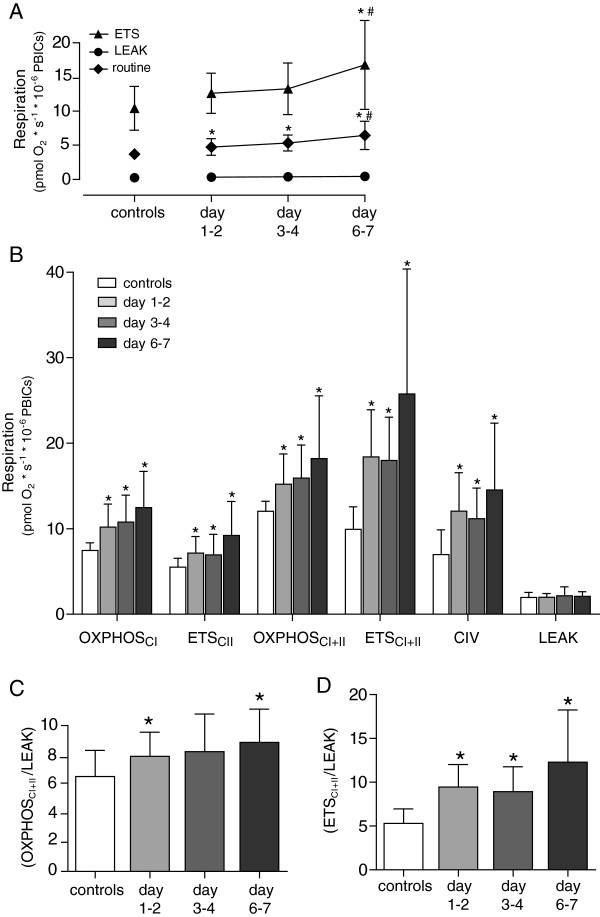
**Cellular mitochondrial respiration.** In **(A)** intact cells and **(B)** permeabilized cells from healthy controls and sepsis patients at three different time points. Control ratios for maximal respiration of **(C)** the oxidative phosphorylation system (OXPHOS) and **(D)** electron-transfer system (ETS). Graphs presented as mean ± SD. *P < 0.05 as compared with controls; ^#^*P* < 0.05 as compared with days 1 to 2. The different respiratory states are explained in the Methods section.

In the first protocol using intact cells analyzed in the patient’s own plasma, routine respiration but not maximal stimulated respiration with FCCP was significantly increased within the first 48 hours of admission (3.7 ± 0.6 versus 4.7 ± 1.2, *P* = 0.04; and 10.4 ± 3.2 versus 12.6 ± 2.5, *P* = 0.06; pmol O_2_ × s^-1^ × 10^-6^ cells, respectively). At the subsequent time points measured, a gradual increase in cellular respiration was noted. At day 6 to 7, routine respiration had increased to 6.4 ± 2.1 pmol O_2_ × s^-1^ × 10^-6^ cells, *P* = 0.001; and FCCP-stimulated respiration was 16.8 ± 6.5 pmol O_2_ × s^-1^ × 10^-6^ cells, *P* = 0.005; Figure 1A. No difference was found in LEAK respiration between sepsis patients and controls or during the different time points measured. Because of the tight coupling of respiration, with LEAK values approaching the residual oxygen consumption, control ratios could not be calculated.

In permeabilized cells, respiration using substrates of either complex I, II, or both for OXPHOS and ETS, as well as substrates of complex IV, all significantly increased compared with controls at days 1 to 2 (10.5 ± 2.4 versus 7.4 ± 0.9, 7.2 ± 1.9 versus 5.5 ± 1.0, 15.6 ± 3.1 versus 12.0 ± 1.2, 18.8 ± 5.3 versus 9.9 ± 2.6, and 12.0 ± 4.5 versus 7.0 ± 2.9 pmol O_2_ × s^-1^ × 10^-6^ cells, respectively; *P* < 0.001 for all comparisons). As with intact cells, a trend was seen to further increase in respiration in the sepsis patients between days 1 and 2 and days 6 and 7; Figure 1B.

Control ratios (CRs) of respiration are calculated as an internal reference of ATP-generating capacity and the extent of control the phosphorylating system exerts on the maximal electron transfer. The control ratios for maximal OXPHOS (OXPHOS_CI+II_/LEAK) and ETS (ETS_CI+II_/LEAK) were higher in patients as compared with controls (except for days 3 to 4 OXPHOS). In addition, in patients with sepsis ETS_CI+II_/LEAK also increased significantly from the first to last time point; Figure 1C, D.

### Cellular mitochondrial content

**Figure 2:**
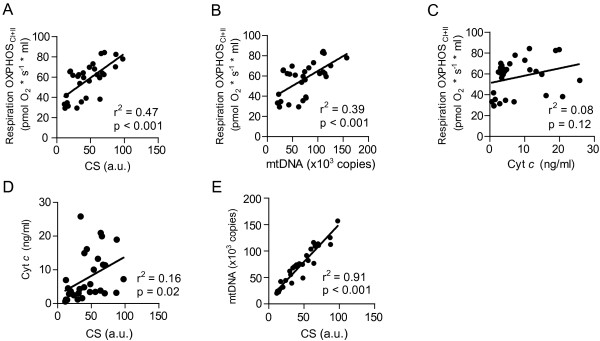
**Correlation between mitochondrial markers. (A)** Citrate synthase (CS), **(B)** mitochondrial DNA (mtDNA), **(C)** cytochrome *c* (Cyt *c)* and maximal ATP-generating respiration in permeabilized cells (OXPHOS_CI+II_). Correlation between CS and Cyt *c***(D)** and CS and mtDNA **(E)**. Correlations were performed by using linear regression.

The septic syndrome is a dynamic event in many ways. Clinically, patients go from being severely ill to either die of the disease or recover. At the cellular level, the subpopulations of PBICs have been shown to change phenotypes and undergo apoptosis [8], and, at the mitochondrial level, sepsis is known to initiate biogenesis [20]. Therefore, to be able to put changes in mitochondrial respiration into context, we analyzed three different mitochondrial-specific markers that were subsequently related to mitochondrial respiration in each sample. CS and mtDNA correlated well to each other as well as to respiration (as shown in Figure 2A, B, E), whereas the correlation for Cyt *c* were weaker compared both to CS and to respiration; Figure 2C, D. No further analysis of Cyt *c* data in relation to respiration was therefore pursued.

**Figure 3:**
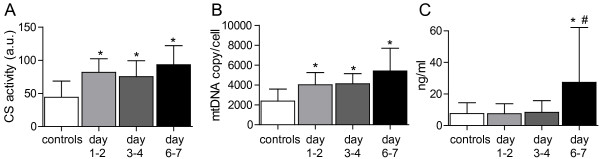
**Levels of mitochondrial markers. (A)** Citrate synthase (CS); (**B)** mitochondrial DNA (mtDNA); **(C)**, cytochrome *c* (Cyt *c)* in analyzed samples from controls and patients during the first week of sepsis. Graphs presented as mean ± SD. *P < 0.05 as compared with controls. ^#^*P* < 0.05 as compared with days 1 to 2; a.u., arbitrary unit.

As displayed in Figure 3 all markers of mitochondrial content increased during the time course studied but with different kinetics. Cyt *c* content remained at the same level as controls during the first two time points measured in the sepsis patients but significantly increased by days 6 to 7 (27.4 ± 35 versus 7.6 ± 6.9 ng/ml; *P* = 0.01). MtDNA displayed a higher copy count per cell as compared with controls at days 1 to 2 (4,030 ± 1,219 versus 2,381 ± 1,217; *P* < 0.001), which remained similar at days 3 to 4 (4,124 ± 1,022) with a further increase, at days 6 to 7, to 5,418 ± 2,281, compared with controls. CS activity was also significantly increased as compared with controls at days 1 to 2 (82 ± 20 versus 44 ± 25 a.u.; *P* < 0.001) and remained similar throughout the other time points (75 ± 24 and 93 ± 29 a.u., respectively).

### Respiration related to specific mitochondrial content

**Figure 4:**
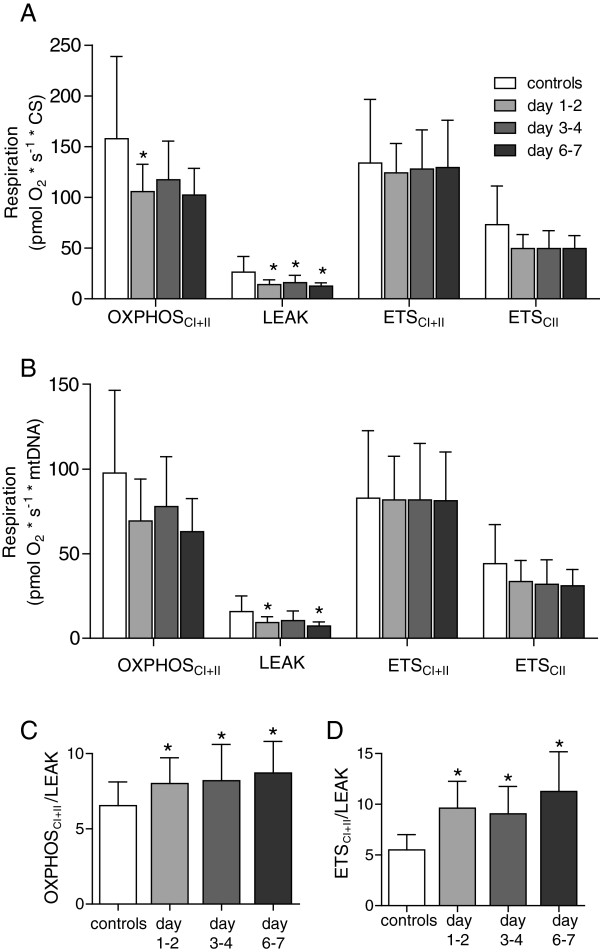
**Adjusted mitochondrial respiration.** Mitochondrial respiration in permeabilized cells adjusted for **(A)** citrate synthase activity (CS) and **(B)** mitochondrial DNA content (mtDNA). The different respiratory states presented are explained in the Materials and methods section. Control ratios for maximal respiration of **(C)**, the oxidative phosphorylation system (OXPHOS), and **(D)** electron-transfer system (ETS) calculated from mtDNA-adjusted respiration. Presented as mean ± SD. *P < 0.05.

CS activity increased relatively more compared with cellular respiration. As a result, mitochondrial respiration, when expressed in relation to CS, displayed a 27% to 52% reduction in OXPHOS_CI+II_ LEAK and ETS_CII_ respiratory states. Despite similar values, the decrease was significant only for maximal ATP-generating respiration at days 1 to 2 and LEAK respiration at all time points. Maximal ETS_CI+II_ capacity, however, remained unaltered (Figure 4A). When related to mtDNA, the trends for OXPHOS and ETS_CII_ were similar (Figure 4B).

However, as shown in Figure 4C and D, the control ratios were not different from those obtained when respiration was expressed per cell. Both OXPHOS_CI+II_/LEAK (controls: 6.5, days 1 to 2: 8.0, days 3 to 4: 8.1, days 6 to 7: 8.7) and ETS_CI+II_/LEAK (controls: 5.5, days 1 to 2; 9.6, days 3 to 4; 9.1, days 6 to 7: 11.2) were significantly increased at all measured time points as compared with controls.

### Respiration related to cytokine levels

Values of the analyzed cytokines, TNF-α, IL-1β, and IL-6, are given in Table 2. All three were significantly increased compared with controls at days 1 to 2. IL-1β returned to similar levels as controls from the second time point, whereas TNF-α and IL-6 displayed gradually decreasing levels throughout the week but remained significantly elevated also at days 6 to 7. No correlation was found between levels of cytokines or dose of catecholamine and mitochondrial respiratory capacity at any of the time points measured (data not shown).

### Alterations in mitochondrial respiration in relation to mortality

**Figure 5:**
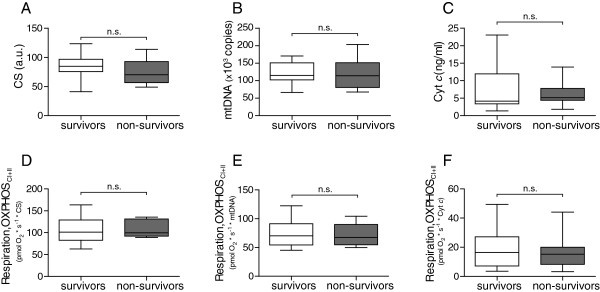
**Differences between survivors and nonsurvivors.** Days 1 to 2 levels of mitochondrial markers **(A-C)** and mitochondrial respiration normalized to respective marker, citrate synthase (CS), mitochondrial DNA (mtDNA), and cytochrome *c* (Cyt *c*) **(D-F)** in 90-day survivors and nonsurvivors. Displayed as box-and-whisker plot (median, IQR and range). Survivors *n* = 13; nonsurvivors, *n* = 7; a.u., arbitrary unit.

In patients with sepsis, previous reports analyzing mitochondrial function within the first days after ICU admission indicated that nonsurvivors have a lower mitochondrial respiratory capacity [10] or mRNA expression of mitochondrial biogenesis factors [12], as compared with survivors. Our results did not reveal any difference between 90-day survivors and nonsurvivors, in either respiratory capacity or markers of mitochondrial content, as measured at days 1 to 2 (Figure 5A to A - F) or any other time point (data not shown).

## Discussion

The key finding of the present study is that PBICs from patients with severe sepsis or septic shock display increased mitochondrial respiratory capacities, as compared with controls, in the early phase of the disease that gradually continued to increase during the course of the first week. The higher cellular respiratory capacity and respiratory control ratios seem to stem from an increase in both mitochondrial quantity and mitochondrial protein content.

We previously showed an increase in mitochondrial respiration in human platelets during the first week of sepsis. This increase was paralleled by a slight increase in Cyt *c* content but unaltered mtDNA [13]. Compared with platelets, PBICs are nuclear-bearing and, as such, have the ability to transcribe and translate nuclear-encoded mitochondrial proteins, without relying on whole cell *de novo* synthesis. In the present study, PBICs also exhibited an increase in respiration throughout the first week of sepsis. Intact PBICs respired primarily by using complex I-linked substrates, as demonstrated by the almost complete cessation of respiration after inhibition of complex I by rotenone (not shown). Also, unaltered LEAK respiration (oligomycin-induced state 4 respiration) in combination with the detected increased parameters of maximal coupled and uncoupled respiration resulted in higher control ratios, confirming an increased efficacy of the mitochondria from the sepsis patients as compared with controls.

In a recent study, the ATP-generating (OXPHOS) capacity of PBICs analyzed with complex II-linked substrates was reduced in the early phase of sepsis, as compared with control subjects that constituted critically ill patients without sepsis. No differences were seen in the other respiratory states (that is, routine, LEAK, or non-ATP-generating respiration (ETS) [10]). In addition, the PBICs of sepsis patients were, as compared with the controls, more sensitive to oligomycin-induced inhibition. This led the authors to conclude that the impairment of mitochondrial function was due to a reduced content of functional complex V [10]. Also, in the sepsis survivors, complex II-linked respiration increased 2.9-fold from day 1 to day 7. Our data agree with this latter finding but stand in contrast to the former, because no inhibition, but rather an increase, of complex II-linked respiration was seen at days 1 to 2. Likely the explanation lies in the difference between the control cohorts. The mean respiration value, with complex II-linked substrates, of PBICs in the critically ill control cohort was approximately double that of the healthy subjects used in the present study. In contrast, the same parameter measured in the sepsis patients was very similar in the two studies. Because any type of inflammation can trigger mitochondrial biogenesis [21], this possibly implies that respiration was upregulated in the critically ill patients used as controls in the study by Japiassu *et al.*[10].

In another study examining mitochondrial respiration in sepsis patients, PBICs, sampled within 48 hours of ICU admission, were found to have elevated basal respiration, most likely due to a proportional increase in LEAK respiration, and inhibited complex II-linked respiration as compared with healthy controls [9]. The difference seen in respiration in relation to the present study is not entirely clear but is possibly explained by the fact that we have measured ETS_CII_ respiration versus OXPHOS_CII_ in the study by Belikova *et al.*[9], again suggesting a difference in complex V function. Also we found that freshly prepared PBICs are prone to aging with consequently relatively rapid deterioration of mitochondrial respiratory function, and a role of the longer incubation time in the study by Belikova *et al.* cannot be ruled out.

To be able to put changes in mitochondrial respiratory function into qualitative and quantitative perspectives, we chose to analyze three completely different mitochondria-specific markers. The enzymatic activity of CS is considered one of the best indicators for mitochondrial matrix content [22,23]. The level of mtDNA has recently been shown to correlate with CS [24], and this was confirmed by our result, in which CS and mtDNA displayed excellent correlation in both the control cohort and the sepsis patients (see Figure 2E). The increase in respiration correlated well with the increase of mtDNA number and CS activity and to a lesser extent with Cyt *c*. We therefore chose to relate mitochondrial respiration to the former two markers. In relation to mtDNA and CS activity, ATP-generating respiration tended to be lower in the sepsis patients as compared with controls. This was shown in a previous study for routine and complex I-linked respiration in PBICs, in which respiration was normalized to protein content [11]. However, as internal quality control, we demonstrated that control ratios from the sepsis patients remained higher throughout the time points measured.

The present data indicate that the initiated process to enhance mitochondrial capacity results in higher mitochondrial numbers per cell, with a net increase in whole-cell respiration. Within each mitochondrion, there seems to be a lower increase of some functional units (for example, complex V), as OXPHOS respiration is less elevated compared with CS and mtDNA, whereas maximal ETS respiration remains constant, in line with recent findings [10]. However, it is important to stress that overall cellular respiration increased in the sepsis population, and the overall functional integrity of mitochondria in PBICs was improved, as evidenced by a lower LEAK and higher control ratios compared with controls at the different time points studied.

The failure to induce a biogenesis response at a transcriptional level in muscle biopsies in patients with critical illness has been associated with worse outcome [12]. Even though our results corroborate the initiation and execution of events leading to increased mitochondrial capacity, we could not detect any difference, at any time point, between survivors and nonsurvivors in mitochondrial respiration or the three different markers of mitochondrial content measured. The roles of muscle and PBICs are completely different in sepsis, which could account for some of the differences seen. Compared with muscle tissue, PBICs have a much higher turnover, and, as immune cells, they go from quiescence to activity during the infectious process and are not subjected to the passivity usually imposed on muscle tissues in sepsis patients. The majority of deaths in the present study occurred within the first week, which precludes drawing any conclusions regarding the change in respiratory capacity for those dying at a later stage. It has been well established that, in sepsis, a decrease of immune cells occurs, especially lymphocytes, as a consequence of increased apoptosis [25]. However, as recently described, concomitant events of both proliferation and apoptosis are likely present in sepsis [26]. Our results, based on a defined amount of cells and mitochondria being analyzed, suggest an intact mitochondrial quality control of the circulating pool of PBICs.

Posttranslational regulation of the mitochondria is increasingly recognized as an important modulator of respiratory capacity. Mitochondria undergo fusion and fission, whereas upregulated fusion proteins lead to increased respiration [27-29]. Complexes of the ETS, as well as a majority of the metabolic pathways in the mitochondria, have been shown to be subjected to regulation by phosphorylation and dephosphorylation [30], and the electron-transfer complexes are incorporated into larger assemblies, known as supercomplexes, or respirasomes, which are proposed to represent the optimal functional units of mitochondrial respiration and ATP production [31]. The role of these events in regulating the demonstrated increase in mitochondrial respiration requires further study.

A limitation of the present study is that it is not possible to draw conclusions regarding its relevance to mitochondrial function in other vital organs, such as liver, kidney, and heart, that are affected in sepsis-induced MOF. Different cell types have different metabolic requirements and work in a variety of milieus with various demands on mitochondrial function. Also, as energy requirement most likely changes during the course of the sepsis event, we do not know if the enhanced mitochondrial respiration observed is adequate for meeting the metabolic demands, or if a relative energy imbalance exists in the cells. It is clear that immune cells from healthy individuals are altered when exposed to infectious stimuli [32], and this is also reflected in our results. Activated PBICs have a higher rate of apoptosis, and as the septic event changes from pro- to antiinflammatory, different subpopulations could be dominant, and this was not analyzed in the present study.

Further to establish the role of mitochondrial biogenesis, we attempted to assess the levels of PGC1α, a key regulatory protein of mitochondrial biogenesis. However, these attempts were hindered by the low abundance of the protein in our samples.

## Conclusions

We demonstrate that, during the first week of sepsis, cellular respiration of PBICs display an increased capacity because of an increased mitochondrial content but also with an increased efficacy of the OXPHOS system. Nonsurvivors displayed the same increase in respiration as the survivors. The present findings argue against mitochondrial respiratory dysfunction, in this type of cell in sepsis, and it seems clear that mitochondrial function has to be related to a variety of specific markers to place results into a relevant context.

## Key messages

• Peripheral blood immune cells exhibit increased mitochondrial respiratory capacity throughout the course of sepsis.

• The increase in respiratory capacity stems from both an increase in mitochondrial mass and increased efficacy of the OXPHOS system.

• No difference in mitochondrial respiratory capacity was observed between survivors and nonsurvivors.

## Competing interests

The authors declare that they have no competing interests.

## Authors' contributions

FS, MH, and EE conceived of and participated in the design of the study. FS and JP recruited the patients. FS and SM performed the respiratory experiments. SM carried out the mitochondrial DNA, citrate synthase, and cytochrome *c* assays. FS drafted the manuscript. All authors read and approved the final manuscript.
